# The gut of the finch: uniqueness of the gut microbiome of the Galápagos vampire finch

**DOI:** 10.1186/s40168-018-0555-8

**Published:** 2018-09-19

**Authors:** Alice J. Michel, Lewis M. Ward, Shana K. Goffredi, Katherine S. Dawson, Daniel T. Baldassarre, Alec Brenner, Kiyoko M. Gotanda, John E. McCormack, Sean W. Mullin, Ariel O’Neill, Gabrielle S. Tender, J. Albert C. Uy, Kristie Yu, Victoria J. Orphan, Jaime A. Chaves

**Affiliations:** 1 Division of Geological and Planetary Sciences, California Institute of Technology, Pasadena, CA 91125 USA; 2 Department of Biology, Occidental College, Los Angeles, CA 90041 USA; 3 Department of Ecology and Evolutionary Biology, Princeton University, Princeton, NJ 08544 USA; 4 Department of Zoology, University of Cambridge, Cambridge, CB2 3EJ England; 5 Department of Biology, University of Miami, Coral Gables, FL 33146 USA; 6 Colegio de Ciencias Biológicas y Ambientales, Universidad San Francisco de Quito, Diego de Robles y Pampite, Quito, Ecuador; 7 Galápagos Science Center, Puerto Baquerizo Moreno, Galápagos Ecuador; 8 School of Environmental and Biological Sciences, Rutgers University, New Brunswick, NJ 08901 USA

**Keywords:** Galápagos Islands, Darwin’s finches, Blood-feeding, Microbiome, *Geospiza*

## Abstract

**Background:**

Darwin’s finches are a clade of 19 species of passerine birds native to the Galápagos Islands, whose biogeography, specialized beak morphologies, and dietary choices—ranging from seeds to blood—make them a classic example of adaptive radiation. While these iconic birds have been intensely studied, the composition of their gut microbiome and the factors influencing it, including host species, diet, and biogeography, has not yet been explored.

**Results:**

We characterized the microbial community associated with 12 species of Darwin’s finches using high-throughput 16S rRNA sequencing of fecal samples from 114 individuals across nine islands, including the unusual blood-feeding vampire finch (*Geospiza septentrionalis*) from Darwin and Wolf Islands. The phylum-level core gut microbiome for Darwin’s finches included the Firmicutes, Gammaproteobacteria, and Actinobacteria, with members of the Bacteroidetes at conspicuously low abundance. The gut microbiome was surprisingly well conserved across the diversity of finch species, with one exception—the vampire finch—which harbored bacteria that were either absent or extremely rare in other finches, including *Fusobacterium*, *Cetobacterium*, *Ureaplasma*, *Mucispirillum*, *Campylobacter*, and various members of the Clostridia—bacteria known from the guts of carnivorous birds and reptiles. Complementary stable isotope analysis of feathers revealed exceptionally high δ^15^N isotope values in the vampire finch, resembling top marine predators. The Galápagos archipelago is also known for extreme wet and dry seasons, and we observed a significant seasonal shift in the gut microbial community of five additional finch species sampled during both seasons.

**Conclusions:**

This study demonstrates the overall conservatism of the finch gut microbiome over short (< 1 Ma) divergence timescales, except in the most extreme case of dietary specialization, and elevates the evolutionary importance of seasonal shifts in driving not only species adaptation, but also gut microbiome composition.

**Electronic supplementary material:**

The online version of this article (10.1186/s40168-018-0555-8) contains supplementary material, which is available to authorized users.

## Background

First introduced to science by Charles Darwin in his diaries from the voyage of the HMS Beagle [1], Darwin’s finches are a classic example of adaptive radiation, the phenomenon by which species diverge from a common ancestor as they adapt to different ecological niches. Divergence of Darwin’s finches from relatives in South America has taken place in the last ~ 1.5 My, when ancestral finches first colonized the islands [2]. Since this time, 19 formally recognized finch species have evolved within this clade—18 in the Galápagos and 1 in the Cocos Islands [3, 4], with major radiations occurring in the last 300 ka [5, 6].

The ecological drivers of adaptation in Darwin’s finches have been extensively studied [3, 7–9]. Diet has long been recognized as a major factor in the adaptive radiation of finches in the Galápagos, as lineages on different islands developed beaks specialized for food sources available on their islands, with selective pressure especially high during the dry season when food sources are most limited [10]. Darwin’s finches, as a group, include plant-matter consumers—the seed-eating ground finches (*Geospiza fortis*, *G*. *fuliginosa*, *G*. *magnirostris*), herbivorous vegetarian finch (*Platyspiza crassirostris*), *Opuntia* nectar- and pollen-specializing cactus finches (*Geospiza scandens*, *G*. *conirostris*)—and insectivorous finches—the sharp-beaked ground finches (*G*. *difficilis*, *G*. *acutirostris*), woodpecker (*Camarhynchus pallidus*), tree finch (*C*. *parvulus*), warbler finches (*Certhidia olivacea*, *C*. *fusca*) [3]—and the curious blood-feeding vampire finches of Darwin and Wolf Islands (*Geospiza septentrionalis*) [4, 11, 12]. The vampire finches are so called because they supplement their diet with blood harvested from Nazca and red-footed boobies (*Sula granti* and *S*. *sula*) during the dry season when resources are scarce on these remote islands, a unique strategy not used by any other species [13–15].

Growing evidence suggests that gut microbes exert a major influence on animal nutrition, health, immunity, and behavior [16–18]. Reciprocally, large-scale sequencing of 16S rRNA of associated gut bacteria and archaea has revealed that gut microbial diversity can be influenced by diet, host morphology, host phylogeny, or environment [16, 19, 20]. Comparisons between gut communities of closely related vertebrates with different diets have yielded a range of patterns. Diet has been shown to be a strong predictor of gut microbial composition in phyllostomid bats [21, 22], dolphins [23], humans [24], and phylogenetically diverse ant-eating mammals [25]. On the other hand, links between host phylogeny and gut microbiota have been reported from bird species [26] and in selected mammals, such as bears, where despite their restricted bamboo diet, pandas maintain a microbiome similar to other bears [27]. Other studies have identified a combination of determining factors, including phylogeny and diet (e.g., in some birds and baleen whales [28–30]) and biogeography and diet (e.g., in recently radiated African cichlids and Galápagos iguanas [31–33]). Crucially, studies have focused on clades that diverged tens of millions of years ago, whereas less is known about the degree of gut microbiome divergence in younger, relatively recent species radiations.

Recent studies in birds have generated important data regarding the composition of the gut microbiome, yet there remains an enormous gap in knowledge of most bird lineages [20], particularly non-domesticated species [34]. The gut microbiome of the domestic chicken [17, 35], folivorous hoatzin [36], various seabirds including penguin [37] and petrel [38], and Passeriformes (the order that includes Darwin’s finches) [39] are all dominated by members of the Proteobacteria, Firmicutes, Bacteroidetes, and Actinobacteria, as well as other, low-abundance phyla. A variety of factors influence the gut microbiome, but the role of diet and environment are thought to be of paramount importance in birds [20], even when controlling for other factors, as shown in fledglings of the brood parasite (*Molothrus ater*), a cowbird that lays its eggs in the nests of heterospecific hosts [40].

Here, we analyzed the gut microbiota of 12 species of Darwin’s finches, sampled across 9 of the 18 Galápagos Islands during both the wet and dry seasons. During the wet season, from January to early June, food is abundant, while food becomes limiting and the birds resort to their “adaptive” diet in the dry season, from July to December [10]. Fecal samples were collected from 114 individual finches, including the two remote populations of vampire finch (*Geospiza septentrionalis*) on Darwin and Wolf Islands. Microbial diversity was screened using Illumina high-throughput sequencing of the 16S rRNA gene. A complementary analysis of stable carbon and nitrogen isotopes of feathers from vampire finches and a subset of others was also performed as an independent means to identify dietary signatures. The diversity in diet and lifestyle of Darwin’s finches and the extensive ecological and genetic knowledge of this avian group make them an attractive study subject to examine how host diet, biogeography, phylogeny, and other environmental factors affect the vertebrate gut microbial community over relatively short evolutionary timescales.

## Results and discussion

### The core gut microbiome of Darwin’s finches

**Fig. 1:**
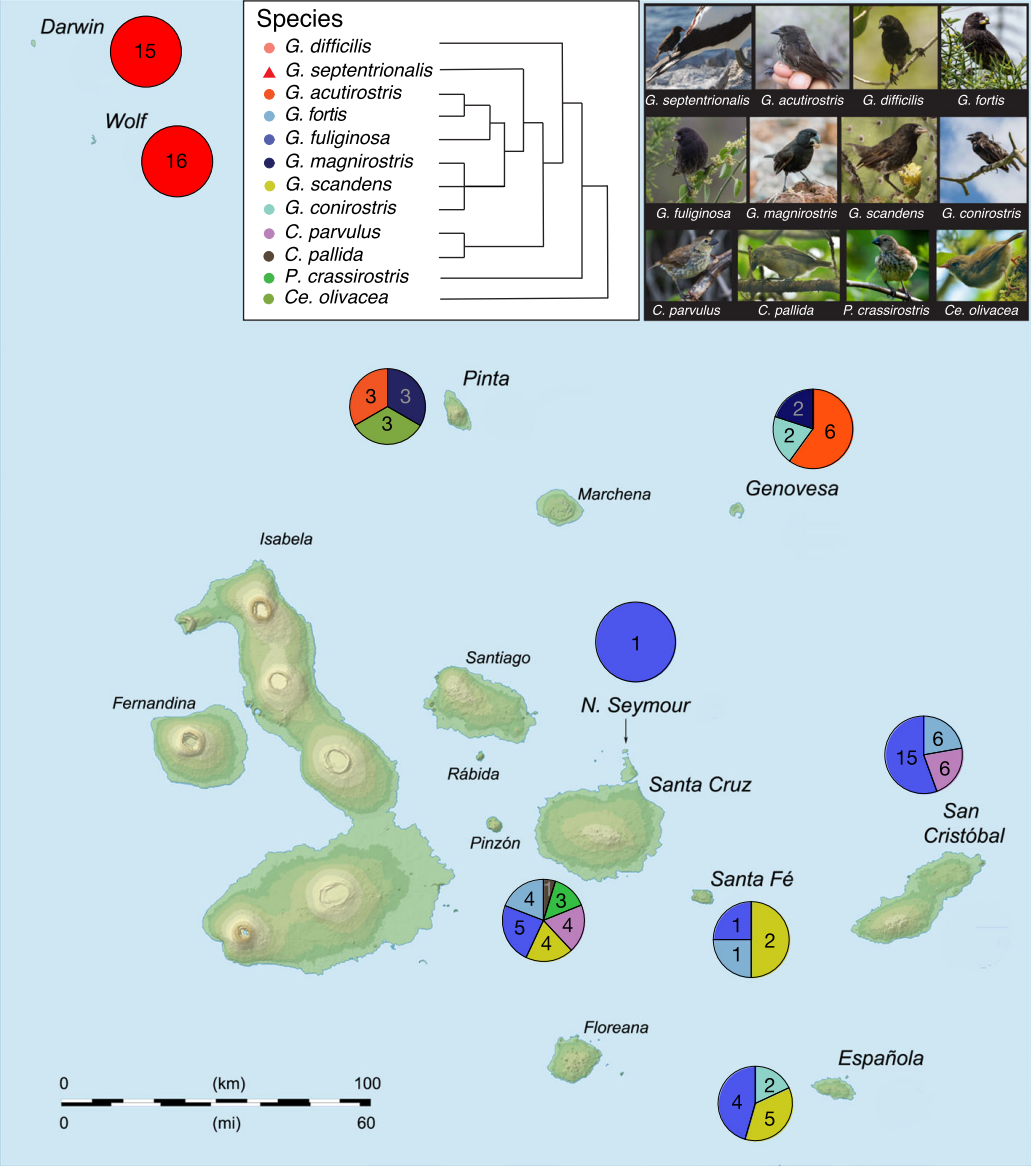
Overview of finch gut microbiome samples collected from the Galápagos Islands. Pie charts represent the number of fecal samples from each species of finch from the different islands colored according to the legend. Cladogram of host finch phylogeny modified from Lamichhaney et al. [5]

**Fig. 2:**
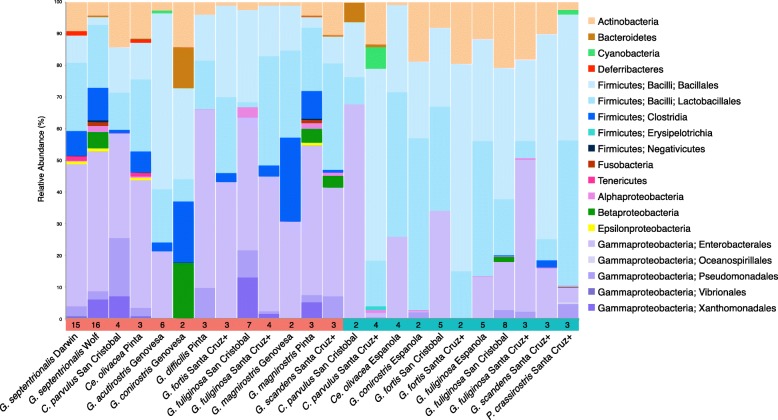
Average taxonomic composition of gut microbial communities of Darwin’s finches, from Illumina 16S rRNA gene surveys, based on OTU clustering at 97% identity trimmed to at least 1% relative abundance in at least one finch. Data is grouped by season, island, and species. The colored bar at the bottom, which also shows sample sizes, distinguishes finch samples from the dry (red) and wet (blue) seasons (Note that for this analysis, Santa Cruz, and neighboring islands Santa Fé, and North Seymour are grouped together as “Santa Cruz +” ). An average of 25,382 reads per finch, comprised of 297 unique OTUs (clustered at 97% similarity level), was recovered at greater than 1% relative abundance in at least one finch across the dataset

The 16S rRNA gene diversity of the gut microbiome of Darwin’s finches was characterized from fecal samples collected from 114 finches, representing 12 of 19 species distributed across nine of the Galápagos Islands (Fig. 1; Table 1; note that as a singleton, sequence data from the one specimen of C. pallida was removed from subsequent analysis). This dataset yielded an average of 25,382 16S rRNA sequences per finch and collectively comprised 297 unique bacterial ribotypes (OTUs; 97% similarity level), present above a minimum threshold set at 1% relative abundance in at least one finch in the dataset. These OTUs primarily grouped within three major bacterial phyla: Firmicutes, Proteobacteria, and Actinobacteria (Fig. 2; Additional file 1: Table S1). The number of OTUs recovered from each species was 71.6 ± 9.8 (avg ± SD; Additional file 2: Table S2). Shannon diversity indices for the Galápagos finch microbiome dataset ranged between 1.4 and 2.2 per finch species (Additional file 2: Table S2), within the range of diversity indices from other avian species (0.6–3.4) [26].

**Table 1:** Finch species sampled in this study, including information about general diet category, sampling island, and number of specimens collected during each of the dry and wet season

Species	Common name	Diet category	Island	No. samples (season)	
				Dry	Wet
*G*. *septentrionalis*	Vampire finch	Carnivorous^2^, inc. blood	Wolf	16	0
			Darwin	15	0
*G*. *acutirostris*	Sharp-beaked ground finch	Insectivorous^3^	Genovesa	6	0
*G*. *difficilis*	Sharp-beaked ground finch	Insectivorous^3^	Pinta	3	0
*G*. *fortis*	Medium ground finch	Herbivorous (seeds)^4^	San Cristóbal	1	5
			Santa Cruz	3	1
			Santa Fé	0	1
*G*. *fuliginosa*	Small ground finch	Herbivorous (seeds)^4^	Española	0	5
			North Seymour	0	1
			San Cristóbal	7	8
			Santa Cruz	4	1
			Santa Fé	0	1
*G*. *magnirostris*	Large ground finch	Herbivorous (seeds)^4^	Genovesa	2	0
			Pinta	3	0
*G*. *conirostris*	Large cactus finch	Herbivorous (*Opuntia* cactus)	Española	0	2
			Genovesa	2	0
*G*. *scandens*	Cactus finch	Herbivorous (*Opuntia* cactus)	Santa Cruz	3	1
			Santa Fé	0	2
*Ce*. *olivacea*	Green warbler finch	Insectivorous	Española	0	4
			Pinta	3	0
*C*. *parvulus*	Small tree finch	Insectivorous	San Cristóbal	4	2
			Santa Cruz	0	4
*C*. *pallida*^1^	Woodpecker Finch	Insectivorous	Santa Cruz	0	1
*P*. *crassirostris*	Vegetarian finch	Herbivorous (leaves)	Santa Cruz	0	3
*Total*	12 Species	–	9 Islands	72	42

Genus abbreviations: *C = Camarhynchus*, *Ce = Certhidia*, *G = Geospiza*, *P = Platyspiza*

^1^The solitary *C*. *pallida* sample was excluded from statistical analyses

^2^During the dry season, *G*. *septentrionalis* eats blood, eggs, scat of the Nazca boobies

^3^Although these finch species are classically identified as seed-eaters, behavioral observations at the time of collection indicated that *G*. *difficilis* on Pinta was foraging on 100% insects in both wet and dry seasons, and *G*. *acutirostris* on Genovesa fed 98% on insects in the wet season, and 80% on insects in the dry season

^4^While these finches predominantly eat seeds, they consume insects on occasion

The analysis of the core microbiome, consisting of OTUs that were universally shared among the 113 finches (after removal of the single *C*. *pallida*), revealed a dominance of Firmicutes (50% average relative abundance, by 16S rRNA recovery), Proteobacteria (40%), and Actinobacteria (8%; Fig. 2; Additional file 3: Figure S1). This pattern has striking resemblance to other avian microbiomes, where surveys of diverse Neotropical birds recovered a similar number of phylotypes (~ 201 bacterial OTUs) belonging to the same three phyla (Proteobacteria, Firmicutes, and Actinobacteria, average relative abundances 46%, 37%, and 1%, respectively) [28]. Two specific OTUs present in every finch included representatives of *Enterobacter* (17% average abundance; Gammaproteobacteria) and *Enterococcus* (13% average abundance; Bacilli). A *Shigella-*affiliated OTU (8% average abundance; Gammaproteobacteria) was also detected in all but one finch from Darwin Island.

Representatives from the Bacteroidetes and five additional phyla (Chloroflexi, Cyanobacteria, Deferribacteres, Fusobacteria, and Tenericutes) were detected, but occurred in only a subset of the finches (typically at < 1% average abundance; Fig. 2; Additional file 1: Table S1). While polysaccharide-degrading Bacteroidetes represent a significant component of the gut microbiome in many vertebrates [16, 38, 41, 42], they were rare, or not detected, in Darwins’ finches (< 0.5% on average in 23/113 finches, and absent in 88 finches), with the exception of 2 finch individuals (*C*. *parvulus*, San Cristóbal, wet season and *G*. *conirostris*, Genovesa, dry season; Bacteroidetes at 12% and 26%, respectively). Decreased Bacteroidetes presence has been previously observed in birds; however, the previously reported relative abundance of 3–20% is comparably higher than the vast majority of the finches analyzed in this study [20, 39, 40]. Even finches specializing in a carbohydrate-rich diet (e.g., the vegetarian and cactus finches, *P*. *crassirostris* and *G*. *scandens*) did not show a positive relationship with this bacterial phylum, with only one *P*. *crassirostris* and one *G*. *scandens* harboring Bacteroidetes OTUs, < 0.02% total microbiota. It is currently unknown which bacterial lineages may occupy a similar niche as Bacteroidetes in the finch microbiome.

### Uniqueness of the vampire finch microbiome

**Fig. 3:**
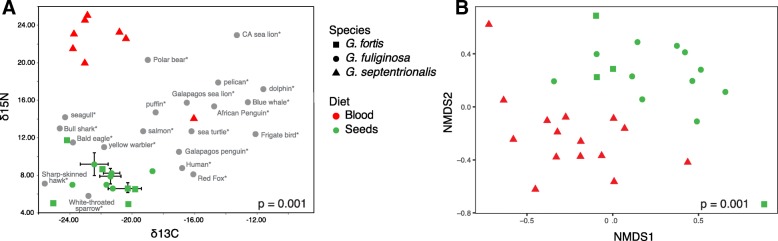
**a** δ^15^N vs. δ^13^C isotopic composition of feathers from Darwin’s finches, specifically the vampire finch (*G*. *septentrionalis* from Wolf Island; red triangles), medium ground finch (*G*. *fortis*; San Cristóbal Island; green squares), and small ground finch (*G*. *fuliginosa*; San Cristóbal Island; green circles), in comparison to other animal tissues (grey dots). For the finches, each symbol represents values recovered from one individual bird. Error bars, where present, indicate the range of values obtained from two to three different feathers from the same individual. δ^13^C for ground finch feathers ranged from − 24.1 to − 18.7‰ vs. PDB, and from − 23.8 to − 16.1‰ vs. PDB for vampire finches. δ^15^N ranges from + 4.9 to + 11.7‰ for ground finches and between + 14.2 and + 25.1 ‰ δ^15^N for vampire finches. The vampire finch feathers differ significantly in δ^15^N (ANOVA *p* < 0.0001), but not δ^13^C (ANOVA *p* > 0.9). Isotopic values from other animals were reported in the following: [44–46, 78–87]. **b** Non-metric multidimensional scaling (NMDS) ordination of three species of gut microbial communities of Darwin’s finches according to diet. Taxonomic (OTU) clustering is at 97% identity and abundance weighted by taking the fourth-root of the OTU relative abundance in each finch

Perhaps the most extreme nutritional strategy among Darwin’s finches is that of the vampire finch, *Geospiza septentrionalis*, endemic to the remote northernmost islands of Darwin and Wolf. During the dry season on the Galápagos Islands, when samples for this study were collected, vampire finches feed primarily on the blood and eggs of boobies (*Sula* spp.; Fig. 1, inset), as well as partially digested fish regurgitate and guano (D. Baldassarre, personal observation, see Additional file 4 for information on finch feeding behavior). By contrast, finches on other islands consume plant-based foods and insects throughout the year [10, 15]. Indeed, the stable isotope values of feathers collected from vampire finches during the dry season were notably enriched in nitrogen-15, with δ^15^N values between + 14.2 and + 25.1‰, significantly different from the two ground finch species, *G*. *fortis* and *G*. *fuliginosa*, δ^15^N = + 4.9 and + 11.7‰, respectively; ANOVA *p* < 0.0001; Fig. 3a. The positively shifted δ^15^N values of the vampire finches are more similar to marine carnivores, including sea lions and polar bears, than to other avian species [43–45]. The distinct δ^15^N values for *G*. *septentrionalis*, consistent with a high trophic-level marine-feeding animal, are likely the outcome of the consumption of proteins sourced predominantly from Nazca boobies, which eat fish. Alternatively, a trend towards positive nitrogen stable isotope ratios has also been shown to occur under starvation conditions, associated with the breakdown of muscle proteins [46]. Given the extreme conditions during the dry season on Darwin and Wolf Islands, this cannot be ruled out as a contributing factor; however, the vampire finches collected in this study had consistently high keel fat content and weights between the wet and dry seasons (J. Chaves, personal observation), suggesting that their distinct isotopic composition is not likely due to nutritional deprivation.

**Fig. 4:**
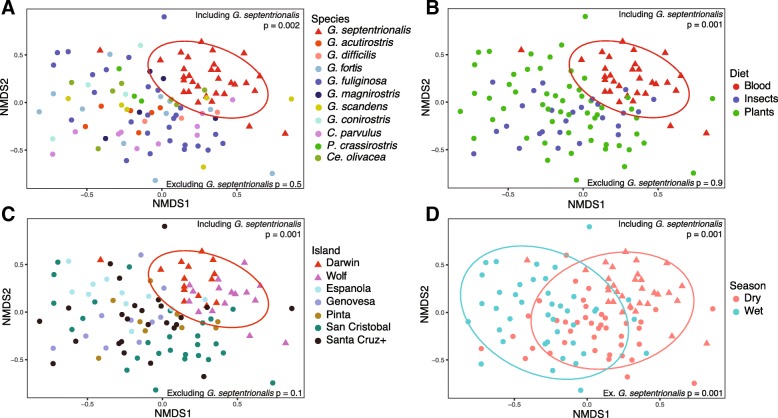
Non-metric multidimensional scaling (NMDS) ordination of gut microbial communities of Darwin’s finches according to **a** species, **b** island, **c** diet, and **d** season for all finches in the sample set. Taxonomic (OTU) clustering is at 97% identity and abundance weighted by taking the fourth-root of the OTU relative abundance in each finch. Ellipses represent 90% confidence windows following a multivariate t-distribution. ANOSIM *p* values are shown for relationships, including and excluding the vampire finch species *G*. *septentrionalis*

While there appears to be strong conservation in the core gut microbiome diversity among Darwin’s finches overall (analysis of similarities (ANOSIM) *p* > 0.05; Fig. 4; Additional file 5: Table S3), vampire finches of Darwin and Wolf Islands are a notable exception (Fig. 4a). Non-metric multidimensional scaling (NMDS) ordination and ANOSIM of Bray-Curtis and UniFrac dissimilarities revealed that the gut microbiomes of the vampire finches clustered separately from all other finches (ANOSIM R 0.23–0.38, *p* = 0.002, Fig. 3b isotope finches, 4A full dataset; Additional file 6: Figure S2A dry season; Additional file 5: Table S3). Notably, the microbiome of the vampire finch, *Geospiza septentrionalis*, is also dissimilar from the other closely related sharp-beaked ground finches on other islands (*Geospiza difficilis sensu lato*: *G*. *difficilis sensu stricto* on Pinta and *G*. *acutirostris* on Genovesa Island; ANOSIM R = 0.57, *p* = 0.001; Additional file 5: Table S3, Additional file 6: Figure S2B).

**Fig. 5:**
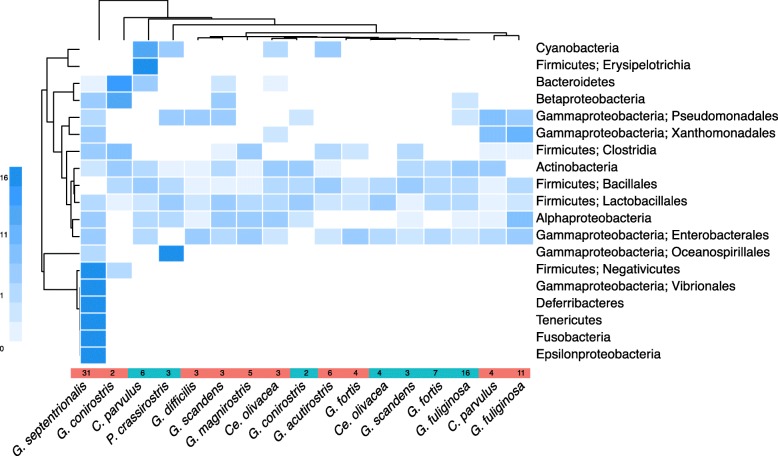
Heatmap showing the weighted average taxonomic composition of the gut microbial communities of Darwin’s finches grouped by season and species. Color represents the deviation from average compositional abundance of bacterial taxa, with 0% compositional abundance as white, average as light blue, below average as paler blue, and above average in dark blue (key at left). The bottom colored bar distinguishes finch samples collected during the dry (red) and wet (blue) seasons, with the sample size indicated for each finch group. Dendrograms group finches and bacterial taxa based on Euclidean distances of the compositional abundance matrix. Note the extreme deviation in the vampire finches: the average vampire finch has 15–17 times more Deferribacteres, Fusobacteria, Tenericutes, Negativicutes, and Epsilonproteobacteria than the average Galápagos finch overall

Carnivorous diets have been shown to be linked to gut microbiome divergence from closely related herbivores [16, 21, 24, 31, 47]. The divergence of the vampire finch microbiome from other finches in our study is attributed to the presence of several lower abundance taxa that were absent or extremely rare in the other finches (Fig. 5). These unique taxa included *Fusobacterium* and *Cetobacterium* (Fusobacteria; Fusobacteriaceae), *Ureaplasma* (Tenericutes; Mycoplasmataceae), *Mucispirillum* (Deferribacteres; Deferribacteraceae), *Campylobacter* (Epsilonproteobacteria; Campylobacteraceae), and various members of the Clostridia (Firmicutes; Clostridiaceae and Peptostreptococcaceae). Two Fusobacteria OTUs accounted for ~ 0.75% of the average vampire finch microbiome, but less than 0.0004% of the relative abundance in the gut microbiomes of other finches (Mann-Whitney *U* test *p* = 0.003). Twenty-five Clostridia OTUs were collectively recovered from several finch species, but were significantly more abundant in the vampire finches (1.8% vs 9.2%; Mann-Whitney *p* = 0.000008). While the specific niche of Fusobacteria and Clostridia in the gut community is not yet known, the prevalence of these organisms in the guts of vultures and alligators [48, 49] suggests a possible relationship to the unique and specific carnivore-like diet of the vampire finch.

Additional taxa that were shown to be statistically different in the vampire finch, relative to other finches, included an OTU affiliated with the genus *Campylobacter* (Epsilonproteobacteria), at 0.9% average relative abundance versus 0.0005% from other Darwin’s finches (Mann-Whitney *p* = 0.0002); two *Ureaplasma* (Tenericutes; Mycoplasmataceae) OTUs at 0.54% (10/31 samples) vs. 0.0001% in the other finches (3/82 samples; Mann-Whitney *p* = 0.004); and one *Mucispirillum* (Deferribacteraceae) OTU, accounting for 0.7% of the average vampire finch microbiome (in 17/31 specimens) versus 0.0002% in others (2/82 samples; Mann-Whitney *p* = 0.000009). The recovered *Campylobacter* OTU was closely related to *Campylobacter volucris*, isolated from gulls [50]. *Campylobacter* have been reported in association with the gut microbiome of some Passeriformes species, as well as other birds, reptiles, and mammals [29, 41], and their distribution and inter-species transmission as pathogens has been well studied [51]. *Ureaplasma* and *Mucispirillum* have also been previously described in association with shorebirds [52], but little is known about their overall distribution and functional role in the host. Given their common occurrence in the vampire finches, it is possible that the acquisition of these lower abundance genera may be linked to the close association of the vampire finch with the co-occurring Nazca and red-footed boobies on Darwin and Wolf Islands.

While other exclusively sanguivorous animals, such as vampire bats and leeches, harbor *Aeromonas* as an obligate gut symbiont [53, 54], this bacterial taxon was not recovered from the vampire finches, possibly due to the non-obligate nature of their blood feeding. However, a recent investigation of the microbiome of the common vampire bat (*Desmodus rotundus*) found only very low abundances of *Aeromonas* sp. (< 0.2%), indicating that *Aeromonas* might not be essential for obligate sanguivory, as previously presumed [22].

Blood feeding in Darwin’s finches was highly distinct from the other diet categories of insectivory and plant-based diets (seeds, *Opuntia* cactus nectar, and leaves) (ANOSIM *R* = 0.23, *p* = 0.001; Fig. 4b). The statistical significance of this difference was maintained after accounting for the larger vampire finch sample size (ANOSIM *R* = 0.38, *p* = 0.003), sampling only in the dry season (ANOSIM *R* = 0.34, *p* = 0.001), as well as focusing specifically on a taxonomically narrow group of finches (previously named *G*. *difficilis*), of which the vampires belong (ANOSIM *R* = 0.57, *p* = 0.001). In the full dataset, the distinctiveness of the vampire finch species (ANOSIM *R* = 0.23, *p* = 0.001) accounts for nearly all of the variation attributable to species differences (*R* = 0.27, *p* = 0.001; Fig. 4a; Additional file 5: Table S3), with species differences absent when the vampire finches are omitted (ANOSIM *R* = 0, *p* = 0.5). By contrast, no discernable difference between insectivory versus plant-based diets was detected in either the dry season (ANOSIM *R* = 0.02, *p* = 0.3) or the wet season (*R* = 0.01, *p* = 0.4), or when considering the full dataset, minus vampire finches (*R* = 0, *p* = 0.9; Additional file 5: Table S3).

Geographic isolation, host phylogeny, and unusual diet are all possible factors influencing the composition of the gut microbial community in the vampire finch. Darwin and Wolf Islands are geographically isolated from the other islands of the Galápagos (~ 300 km from Santa Cruz, more than double the distance between all other Galápagos Islands), thus limiting genetic exchange with finches within the archipelago [4]. Targeted comparison of the gut microbiome of vampire finches from either Darwin or Wolf Island, separated by a mere 40 km, found them to differ significantly from each other (ANOSIM *R* = 0.16, *p* = 0.001; Additional file 6: Figure S2C, Additional file 5: Table S3), due to differences in the abundance of minor taxa (Figs. 2 and 5). This suggests that geographic separation may be a factor influencing the gut microbiome. However, preliminary analysis of a more limited sample set across the remaining islands did not identify a trend. For example, an analysis of *G*. *fuliginosa* and *G*. *fortis* did not reveal a strong divergence across the islands of San Cristóbal and Santa Cruz (~ 65 km apart; ANOSIM *R* = 0.06, *p* = 0.15). Deeper sampling of individual species across islands will be necessary to more confidently examine the potential role of island biogeography.

Additionally, finch phylogeny appeared insignificant for the deepest-sampled non-vampire finches (*G*. *fuliginosa* and *G. fortis*, *n* = 11 each; ANOSIM *R* = 0, *p* = 0.48), suggesting that host phylogeny is not likely a major influence on the gut microbiome of Darwin’s finches. Additional sampling from more distantly related and under-sampled species is necessary to thoroughly clarify the role of phylogeny. Nevertheless, of these potential variables examined, a blood-feeding diet of *Geospiza septentrionalis*, independently supported by isotopic evidence, appears to be the significant contributor to the unique diversity of the gut microbiome.

### Season has a significant influence on the gut microbiome of Darwin’s finches

Investigations of Darwin’s finches provided a unique opportunity to compare the influence of extreme seasonal shifts on the gut microbiome. The Galápagos Islands are well known for strong seasonal cycles driving the availability of vegetation and other food sources utilized by Darwin’s finches, and it is during the dry season that natural selection for unique feeding adaptations is most intense [3, 55]. It is thought that food limitation during the dry season induces specialization and speciation of the various finch species, as species adapt to recalcitrant resources such as *Tribulus* seeds, a process documented in Darwin’s ground finches especially during extreme El Niño events [3, 10]. Plant growth and seed production occurs during the wet season (January to June), which is also associated with an increase in arthropod abundance [7]. During the dry season, however, finches undergo significant dietary stress as plant-based food sources become limiting [55] and it is then that speciation becomes observable [3]. Season has been identified as a significant driver of gut microbiome divergence in other animals, including alligators, which engage in protracted fasting during the winter [48]. Whether the microbiome of Darwin’s finches responds to seasonal extremes on the Galápagos Islands is an outstanding question.

Using our dataset consisting of 72 individuals from the dry season and 41 from the wet season, we found season to be significantly associated with differences in microbial composition. Statistical differences in the finch gut microbiome between the wet and dry seasons occurred across the entire dataset (ANOSIM *R* = 0.27, *p* = 0.001; Fig. 4d), as well as when vampire finches were excluded (ANOSIM *R* = 0.17, *p* = 0.001). Seasonal differentiation was also detected strictly within the two finch species sampled during both seasons (*G*. *fuliginosa* and *G*. *fortis*) (ANOSIM *R* = 0.28, *p* = 0.001; Additional file 6: Figure S2D, Additional file 5: Table S3). Members of the Gammaproteobacteria were the primary driver of the variation in finch microbiome during the dry season (compositional abundance 48% in dry vs. 21% in wet, Mann-Whitney *U* test *p* = 0.01), while Bacilli were dominant during the wet season (35% in dry vs. 65% in wet, Mann-Whitney *p* = 0.00008) (Fig. 2). Higher relative abundances of Actinobacteria were also documented in the wet season (6% vs. 12%), although this difference was not significant (Mann-Whitney *p* = 0.5).

A significant shift in the finch gut microbial community with season may reflect temporal shifts in response to changes in weather, food resource availability, or other factors. The seasonal variation in the gut microbiome among all granivorous/herbivorous finches (*G*. *magnirostris*, *G*. *fortis*, *G*. *fuliginosa*, *G*. *scandens*, *G*. *conirostris*, *P*. *crassirostris*) supports the ecological observations of finches optimizing their feeding strategies by season (ANOSIM *R* = 0.21, *p* = 0.001; *n* = 50). Omnivorous/insectivorous finches (*G*. *difficilis*, *G*. *acutirostris*, *C*. *parvulus*, and *Ce*. *olivacea*), on the other hand, were not significantly different by season (ANOSIM *R* = 0.08, *p* = 0.2; *n* = 20), consistent with lesser shifts in types of foodstuffs between seasons. It is conceivable, however, that either hormonal or ecological changes coincident with season cause the microbiome to shift in composition, irrespective of diet. For instance, when the finches nest during the wet season, they may come into contact with different microorganisms, such as those carried by the invasive nest parasite, *Philornis downsii* [56]. Firmicutes and Proteobacteria were recently reported as the dominant taxa in the microbiome of *P*. *downsii* on Santa Cruz Island [57].

A preliminary assessment of diet specialization in a limited number of these finches (broadly categorized as granivory, omnivory, insectivory, nectarivory, and herbivory) revealed no obvious relationship with the gut microbiome, despite well-documented shifts in finch beak morphology and behavior. Other bird species have shown similar patterns, where only the most extreme diets (e.g., vultures, and other scavengers) resulted in a marked difference in the microbiome [20]. Most of Darwin’s finch species have remained omnivorous, with an emphasis on seeds; thus, the influence of diet specialization on the gut microbiome in these species is expected to be minor compared to other animal groups where more extreme dietary specialization has led to shifts in composition (e.g., phyllostomid bats [21, 22] and cichlid fishes [31]). Additional sampling—particularly of the seed-specializing ground finches, *G*. *fuliginosa*, *G*. *fortis*, and *G*. *magnirostris*, which are distributed across multiple islands, as well as dietary specialists such as the vegetarian *P*. *crassirostris* and insectivorous *Ce*. *olivacea*—may reveal more subtle trends driving gut microbiome composition.

## Conclusions

Darwin’s finches have long been recognized as a model system for investigating interactions between biogeography, dietary specialization, morphology, and other aspects of evolutionary radiation. To this list can now be added the interaction of these factors with the composition of the gut microbial community. The radiation of Darwin’s finches has occurred relatively rapidly and recently (~ 300 ka). During this time, it appears that the gut microbial composition of most finch species has remained conserved among the majority of species examined in this study. A conspicuous exception to this conservation occurs in the case of the vampire finch, *Geospiza septentrionalis*, with a microbial community significantly distinct from the other finches. Because of the barrenness of Darwin and Wolf Islands, the vampire finches undergo extreme dietary limitations during the dry season, thus leading to blood feeding [15]. Their unique and carnivore-like diet likely contributes to the distinct gut microbial composition, suggesting that cases of extreme dietary divergence can overpower phylogenetic inertia to drive shifts in microbiome composition, even over short evolutionary timescales. Future studies in other recent avian radiations that include dietary extremes, such as the blood-feeding Galápagos Hood mockingbird (*Mimus macdonaldi*), should help with investigating this hypothesis.

A robust seasonal difference was observed between finches collected during the wet and dry seasons and may reflect temporal shifts in response to changes in food resource availability, weather, mating or nesting, or other factors. Further sampling—particularly resampling individual finches through time, across seasons—may help to resolve these uncertainties. It will be interesting to see whether the trends observed in this year-long wet-to-dry cycle is reflected in longer term datasets or with more extreme weather periods experienced, for example, during El Niño/La Niña conditions. This goal will be challenging given their remote nature, enhanced diversity, and numerous overlapping influences. Regardless, Darwin’s finches continue to capture our attention and reveal new secrets after more than a century and a half of study.

## Methods

### Sample collections

Individuals (*n* = 114) from 12 species of Darwin’s finches, from nine islands over both the wet and dry seasons, were captured in mist nets and their fecal material sampled (Table 1, Fig. 1). Dry season samples were collected during November–December 2015, while wet season samples were collected between March and June 2016. Fecal samples were collected by placing the bird in a paper bag on top of a metal mesh grate above a sterilized weigh boat for 3-5 min [58]. Samples were immediately transferred into ~ 2 ml of LifeGuard Preservation Solution (MoBio Laboratories, Carlsbad, CA, USA). Feather samples from three species (*G*. *fortis*, *G*. *fuliginosa*, and *G*. *septentrionalis*) were collected for isotopic analysis in Jan 2016 (Fig. 3a).

### DNA extraction and microbial community analysis

Prior to extracting DNA, samples were centrifuged to remove the LifeGuard preservation solution. Genomic DNA was then recovered from the pelleted fecal material using a PowerSoil DNA isolation kit (MoBio Laboratories, Carlsbad, CA, USA) following the manufacturer’s instructions, with the exception of the addition of a bead-beating step using a Fast Prep 120 instrument; Thermo Electron Corporation). The V4 region of the 16S rRNA gene was PCR-amplified from each extract using the archaeal and bacterial targeted primer set 515F and 806R [59], following the protocol outlined by Case et al. [60]. Successful PCR amplifications were pooled, in duplicate, and barcodes were added according to the Earth Microbiome Project protocol [59, 61, 62]; 5 μl of the amplicon product from the first PCR was used as template in a 5-cycle, 25-μl reconditioning reaction with the same EMP-recommended conditions and the full EMP primers. Samples were mixed together in equimolar amounts and purified in bulk through a Qiagen PCR Purification kit. At all PCR steps, amplification success and purity was checked by gel electrophoresis. Paired-end sequences (2*x* 250 basepair) were generated from barcoded amplicon products at Laragen, Inc. on an Illumina MiSeq platform. At Laragen, the raw data was passed through a filter which demultiplexed the library into individual samples and removed any sequences which had > 1 basepair mismatch on the 12-basepair barcode sequence, and assigned quality scores to each basepair call on every sequence. At the same time, adapter, barcode, and primer sequences were removed. Raw reads were deposited and are available through the Sequence Read Archive under accession number SRP130314.

Sequence data was processed with both the DADA2 pipeline for unclustered high-resolution exact sequence variants (SVs) [63] and QIIME version 1.8.0 [64] for operational taxonomic units (OTUs) at the 97% similarity level. In QIIME, raw sequence pairs were joined and quality-trimmed using the default parameters. Sequences were clustered into de novo OTUs using UCLUST open reference clustering protocol [65]. Then, the most abundant sequence was chosen as representative for each de novo OTU [66]. Taxonomic identification for each representative sequence was assigned using the Silva-119 database [67]. For downstream analyses, the QIIME 97% OTU dataset was trimmed to only include those OTUs representing at least 1% of the total gut community of at least one finch (Additional file 1: Table S1). DADA2 exact sequence variants were calculated using the published pipeline tutorial (v1.4) in R and were analyzed the same as QIIME results, without the 1% abundance cutoff (errors are already accounted for in the DADA2 error model [63]).

Sequences from the 16S rRNA region were aligned using MAFFT [68] and a phylogeny constructed using FastTree [69]. Alpha diversity was estimated using the Shannon index, Simpson index, Fisher alpha, and Chao1 richness and by rarefaction. Diversity statistics were calculated in “phyloseq” v-1.25.2 [70] and are reported at the 97% OTU level in Additional file 2: Table S2. Since results across phylotype extraction methods were highly similar, all in-text figures, values, and statistics are derived from the 97% OTU dataset.

To assess the influence of various factors on the microbiome, the 114-finch dataset was divided 19 different ways, each subdivision accounting for one or more of six different ecological and biological factors. One individual (*C*. *pallida*) was removed from all analyses because it was the sole representative of its species, leaving 113 samples for beta diversity analysis. Each division of the dataset was balanced in sampling within the factor in question, unless otherwise noted. Where category sample sizes differed, the data subdivision was randomly subset by each factor category down to the sample size of the smallest category, with statistics taken from the average of 10 random subsets, as described in Additional file 7: Table S4. The influence of sampling season, island, finch diet, finch species, and the extreme diet of sanguivory were assessed using these subdivisions (Additional file 7: Table S4).

Statistical analysis of the role of each factor in shaping finch microbiome beta diversity was done within each data subdivision on relative (compositional) abundance of each OTU in each finch using non-metric multidimensional scaling (NMDS) ordination plots, analysis of similarities (ANOSIM) of both Bray-Curtis and UniFrac dissimilarities, and PERMANOVA (Adonis) of both Bray-Curtis and UniFrac dissimilarities. Dissimilarity matrices derived from both unweighted (presence-absence) and weighted taxonomic relative abundance (scaled to the fourth-root) were calculated for data from each finch. Summary statistics for all data subdivisions, dissimilarities, and approaches listed above can be found in Additional file 5: Table S3. For data subdivisions where statistical balance was achieved via sub-setting one or more categories within a factor, random subsets were run 10 times and the summary statistics reported represent the average over each run.

The contribution of individual OTUs to statistically significant grouping by factor was queried using similarity percentage (SIMPER), and the significance of differential abundance in individual OTUs by factor grouping were statistically tested using two-tailed Mann-Whitney *U* tests. In an approach similar to beta diversity tests, for data subdivisions where random sub-setting was necessary to achieve equal sample sizes within each factor category, Mann-Whitney tests were run over 100 random subsets of the larger category size, and the average *p* value was reported. Summary statistics and plots were generated in R using the packages “phyloseq,” “vegan,” “ggplot2,” and “RColorBrewer” [70–74].

### Carbon, nitrogen, and sulfur isotope analysis of feathers

The isotopic signature of an organism—including that of δ^13^C, δ^15^N, and δ^34^S of proteins in animal tissues such as hair or feathers—is primarily influenced by food source and is frequently used to discern differences in diet (e.g., [75]). Feathers were collected from a subset of finch species during the transition between dry and wet seasons (Jan 2016). Prior to isotopic analysis, feathers were immersed in 2:1 dichloro-methane:methanol to remove surface oils as described by Blight et al. [76]. After air-drying, feather length and weight were recorded and then individual feathers were split along the rachis and transferred to tin capsules (0.3–1.0 mg), for parallel carbon, nitrogen, and sulfur isotope analysis. Feather δ^13^C and δ^15^N and weight percent C and N (wt.% TOC and TON) were determined via continuous flow (He; 100 ml/min) on a Costech Instruments Elemental Combustion System model 4010 by oxidation at 980 °C over chromium (III) oxide and silvered cobalt (II, III) oxide followed by reduction over elemental copper at 650 °C. CO_2_ was subsequently passed through a water trap and then a 5-Å molecular sieve GC column at 50 °C to separate N_2_ from CO_2_, which was diluted with helium in a Conflo IV interface/open split prior to analysis. Fast jump was calibrated and applied to measure both CO_2_ and N_2_ in the same run. δ^13^C and δ^15^N values were measured on a Thermo Scientific Delta V Plus irMS. δ^13^C and δ^15^N values were corrected for sample size dependency and then normalized to the VPDB scale with a two-point calibration [77]. Error was determined by analyzing sucrose (NIST 8542), acetanilide (Costech Analytical Technologies Inc.), and nitrate (IAEA-NO-3) in combination with in-house standards (from − 45.93‰ to − 10.45‰ for C and − 3.02‰ to 4.70‰ for N). For C measurements, accuracy was ± 0.18‰ (*n* = 50) and precision was ± 0.23‰ (*n* = 50; 1*σ*). For N measurements, accuracy was ± 0.22‰ (*n* = 36) and precision was ± 0.43‰ (*n* = 36; 1*σ*). Feather δ^34^S and weight percent S (wt.% TOS) was measured using continuous flow (He; 120 ml/min) on a ThermoQuest NC2500 mass spectrometer (ThermoQuest Italia, Milan, Italy) by oxidation at 1000 °C over tungsten (VI) oxide and elemental copper. SO_2_ was subsequently passed through a water trap and then a 5-Å molecular sieve GC column at 85 °C. A Conflo III interface/open split was used to introduce SO_2_ to a Thermo Scientific Delta plus XL irMS. The δ^34^S values were corrected for sample size dependency and the normalized to the VCDT scale with a two-point calibration. Error was determined by analyzing two silver sulfides (IAEA-S-2 and IAEA-S-3) in combination with in-house standards including pyrite and sulfanilamide. Accuracy on the δ^34^S measurement was ± 1.84‰ (*n* = 20) and precision was ± 0.35‰ (*n* = 20; 1*σ*).

## Additional files

Additional file 1: **Table S1.** Taxonomic composition of Galápagos finch gut microbial communities, from Illumina 16S rRNA gene surveys, based on OTU clustering at 97% identity trimmed to at least 1% relative abundance in at least one finch. (XLSX 196 kb)

Additional file 2: **Table S2.** Microbiome diversity averages for all finch species measured in this study, along with sample sizes and diet category. Data is based on 16S rRNA gene OTU clustering at 97% identity trimmed to at least 1% relative abundance in at least one finch. (DOCX 71 kb)

Additional file 3: **Figure S1.** Barplot showing the compositional (relative) abundance of microorganisms defined at the phylum to family taxonomic scale in the fecal samples of 113 individual finches in this study. At top, the colored bar distinguishes finch samples from the dry (red) and wet (blue) seasons. An average of 25,382 reads per finch, comprised of 297 unique OTUs (clustered at 97% similarity level), were recovered at greater than 1% relative abundance in at least one finch across the dataset. (PDF 168 kb)

Additional file 4: Additional information regarding the observations of finch feeding behavior, core microbiome results, and microbiome differences between vampire finches on Darwin and Wolf islands. (DOCX 20 kb)

Additional file 5: **Table S3.** Analysis of similarities (ANOSIM) and PERMANOVA (Adonis) significance of each grouping within each dataset subdivision (as described in the Methods section) for weighted (to the 4^th^-root) and unweighted (presence-absence) relative abundance. OTUs were calculated in QIIME at 97%. Significant statistics (*p* < 0.05) are highlighted in yellow. Adonis models used the sample size-balanced variable first when multiple variables were tested. Bars in cells indicate that variance-partitioning was impossible, often due to the factor overlapping with the first factor (ex. In 12c, latitude overlaps heavily with island, with island explaining all and more of the variation attributable to latitude). Dataset subdivisions labeled with a ** indicate small sample size; results are listed as they may show a contrasting trend requiring further investigation. Grayed-out regions in the table are either untestable because the dataset subdivision only has one category in that factor (dark gray) or were not tested because they are redundant with other data subdivisions that have better balance or sample size (light gray). Data subdivisions are color-coded by the factor tested (green = season, blue = diet, red = vampire, yellow = island, purple = species). (PDF 129 kb)

Additional file 6: **Figure S2.** Non-metric multidimensional scaling (NMDS) ordination of Galápagos finch gut microbial communities according to (A) species, during the dry season only (B) island, for the vampire finch only (C) diet, for all medium ground finches, formerly classified as *G. difficilis*, and (D) season, for only *G. fortis* and *G. fuliginosa*. Island was not significant, even when grouping Santa Cruz with close neighbors to boost sampling power (ANOSIM *p* = 0.2), thus in controlling for species and island, season is still significant. Taxonomic (OTU) clustering is at 97% identity and abundance weighted by taking the fourth-root of the OTU relative abundance in each finch. Ellipses represent 90% confidence windows following a multivariate t-distribution. (PDF 909 kb)

Additional file 7: **Table S4.** Description of dataset subdivisions for analysis of individual factors without confounding factors, taken by randomly sub-setting a sub-category of the dataset subdivision where appropriate, as indicated. The influence of season was analyzed using data subdivisions 1 and 3-8, thereby controlling for different species/island/diet groups and the singularity of the vampire finches. Diet was assessed with dataset subdivisions 9-12 by comparing all finch species sampled with the exception of the vampire finches binned into diet categories of herbivorous (including seeds, cactus, and plant matter) and omnivorous (including insects), with control for season and island embedded in the various subsets. Seed-eaters are not generally considered herbivores, thus these groupings were designed broadly to encompass plant matter versus other dietary foodstuffs. The uniqueness of sanguivory was tested in dataset subdivisions 13-15, where season, sample size, and phylogeny (through nearest relatives) were considered as well as a comparison with other finches whose feather isotopes were measured. The role of finch species grouping was analyzed in dataset subdivisions 3, 7, 12, 16, 18, and 19 to remove the confounding influence of the extreme diet of *G. septentrionalis* and to extract the largest groupings while avoiding potential biogeographical (island) and season bias and to assess the ground finches alone. The influence of island was assessed in dataset subdivisions 1-3, 6, 7, 12, and 17-19, with Santa Cruz, Santa Fé, and North Seymour sometimes grouped together as “Santa Cruz +” since these islands are in close geographic proximity (Fig. 1). In some cases (dataset subdivisions 1, 2, and 12), latitude was assessed, as the northern, more isolated islands of Pinta, Genovesa, Wolf, and Darwin versus the remaining islands of the Galápagos. (DOCX 135 kb)
