# Supplementary material for: The gut of the finch: uniqueness of the gut microbiome of the Galápagos vampire finch
Source: Microbiome. 2018 Sep 19;6:167. doi: 10.1186/s40168-018-0555-8 (PMC6146768; doi:10.1186/s40168-018-0555-8)
Supplement: Supplementary file 2 — Table S2. Microbiome diversity averages for all finch species measured in this study, along with sample sizes and diet category. Data is based on 16S rRNA gene OTU clustering at 97% identity trimmed to at least 1% relative abundance in at least one finch. (DOCX 71 kb) [file 40168_2018_555_MOESM2_ESM.docx]

Table S2: Microbiome diversity averages for all finch species measured in this study, along with sample sizes and diet category

| **Species^1^** | **Diet^2^** | **# OTUs^3^** | **Shannon** | **Simpson** | **Chao1** | **Fisher** | **n** |
| --- | --- | --- | --- | --- | --- | --- | --- |
| *C. parvulus* | insects | 67.0 | 2.0 | 0.7 | 90.4 | 8.3 | 10 |
| *Ce. olivacea* | insects | 54.7 | 1.4 | 0.6 | 73.7 | 6.5 | 7 |
| *G. acutirostris* | seeds | 81.0 | 2.0 | 0.7 | 110.1 | 10.7 | 6 |
| *G. difficilis* | seeds | 72.0 | 2.0 | 0.8 | 92.6 | 9.4 | 3 |
| *G. fortis* | seeds | 56.5 | 1.6 | 0.6 | 79.7 | 7.0 | 11 |
| *G. fuliginosa* | seeds | 63.6 | 1.7 | 0.7 | 87.8 | 7.8 | 27 |
| *G. magnirostris* | seeds | 73.0 | 1.7 | 0.6 | 91.1 | 9.9 | 5 |
| *G. scandens* | *Opuntia* | 75.3 | 2.2 | 0.8 | 91.0 | 9.6 | 6 |
| *G. conirostris* | *Opuntia* | 76.3 | 2.1 | 0.8 | 100.5 | 9.7 | 4 |
| *G. septentrionalis* | blood | 76.8 | 2.2 | 0.8 | 94.5 | 10.3 | 31 |
| *P. crassirostris* | plants | 74.0 | 1.5 | 0.5 | 88.2 | 9.1 | 3 |
| *C. pallida*^4^ | insects | 89.0 | 2.1 | 0.7 | 106.1 | 13.3 | 1 |
| All finches | -- | 71.6 | 1.9 | 0.7 | 92.1 | 9.3 | 114 |

**^1^**Genus abbreviations: *C = Camarhynchus, Ce = Certhidia, G = Geospiza, P = Platyspiza.*

^2^Diet category: main diet during the season in which they were collected.

^3^Number of OTU per bird, based on 97% similarity

^4^*C. pallida* was removed from downstream beta diversity analysis because there was only one sample.
