# Supplementary figures and images for: The gut of the finch: uniqueness of the gut microbiome of the Galápagos vampire finch
Source: Microbiome. 2018 Sep 19;6:167. doi: 10.1186/s40168-018-0555-8 (PMC6146768; doi:10.1186/s40168-018-0555-8)

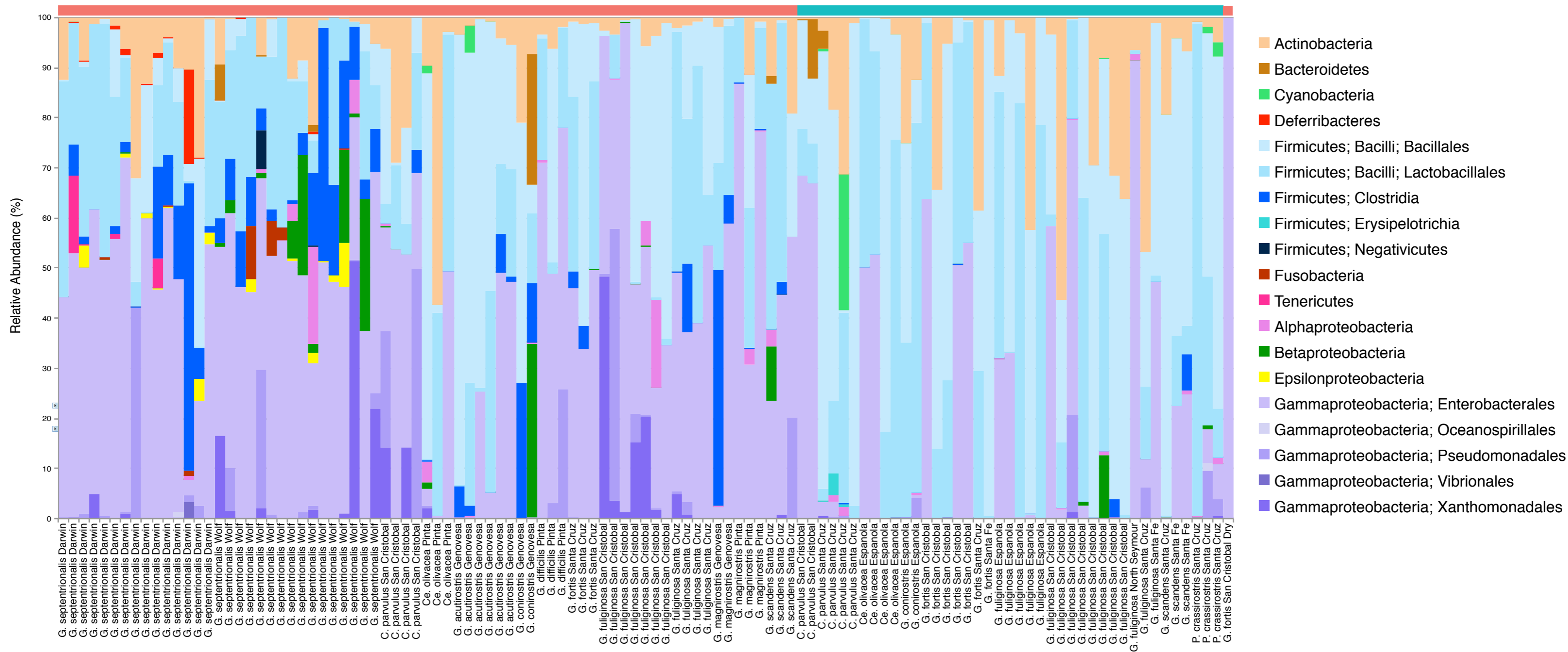

Supplement: Supplementary file 3 — Figure S1. Barplot showing the compositional (relative) abundance of microorganisms defined at the phylum to family taxonomic scale in the fecal samples of 113 individual finches in this study. At top, the colored bar distinguishes finch samples from the dry (red) and wet (blue) seasons. An average of 25,382 reads per finch, comprised of 297 unique OTUs (clustered at 97% similarity level), were recovered at greater than 1% relative abundance in at least one finch across the dataset. (PDF 168 kb) [file 40168_2018_555_MOESM3_ESM.pdf]

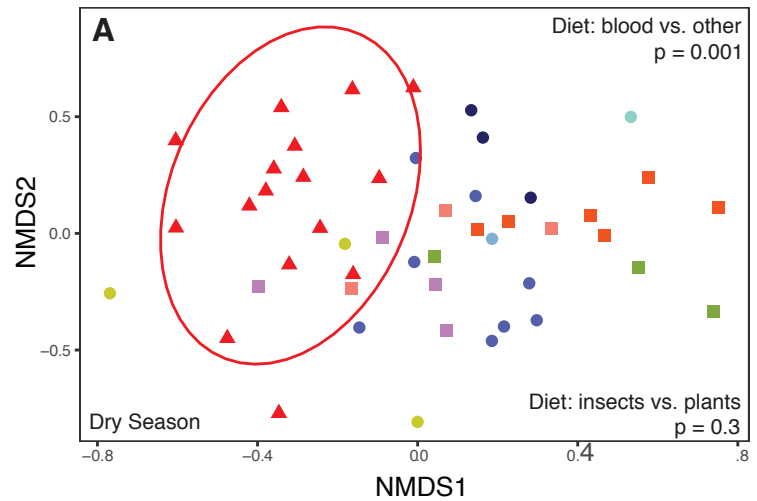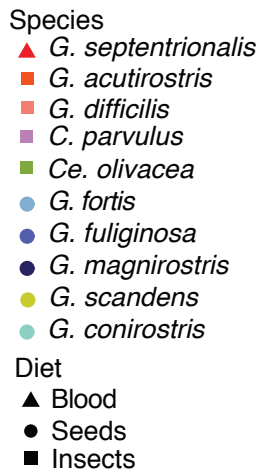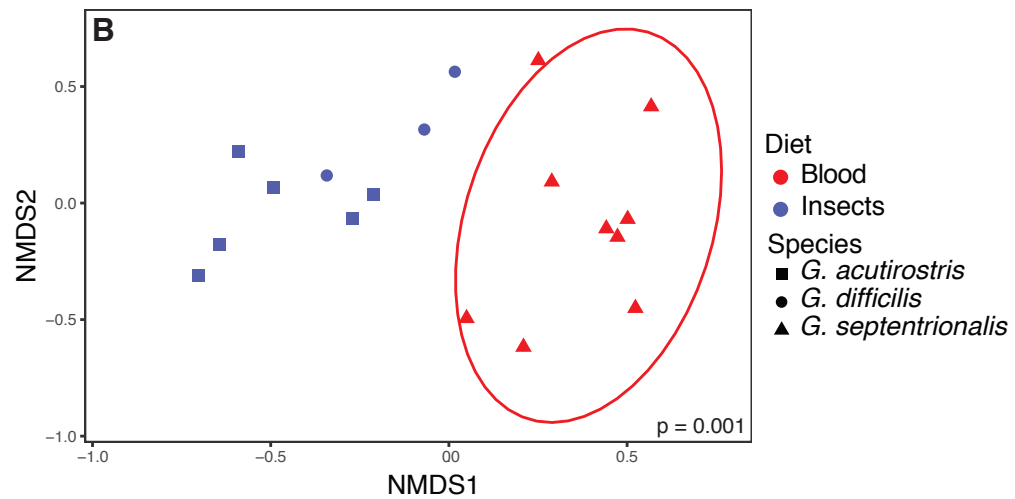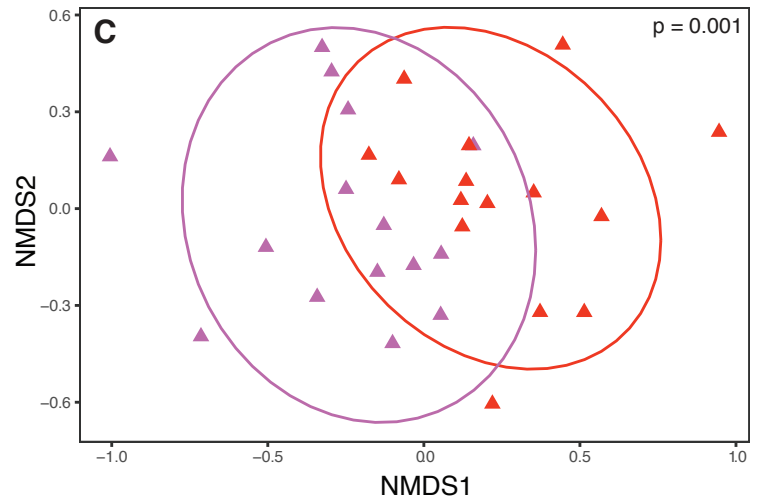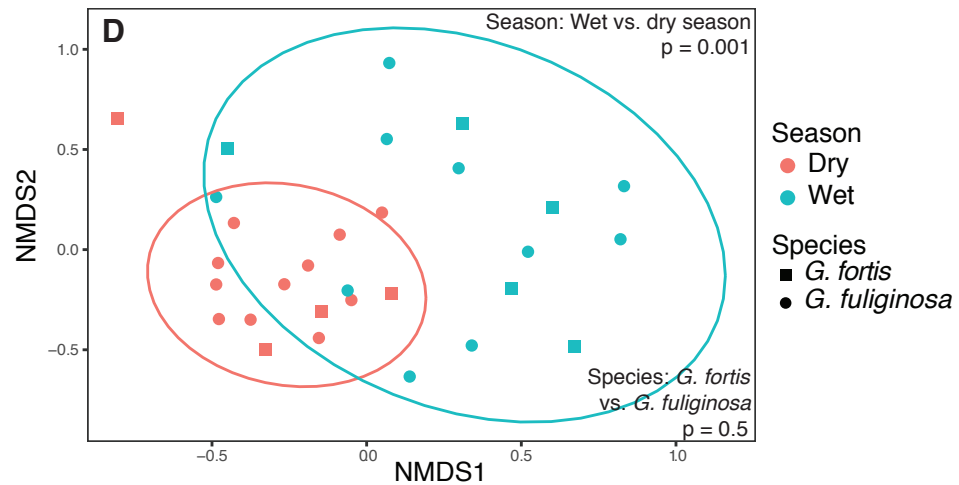

Supplement: Supplementary file 6 — Figure S2. Non-metric multidimensional scaling (NMDS) ordination of Galápagos finch gut microbial communities according to (A) species, during the dry season only (B) island, for the vampire finch only (C) diet, for all medium ground finches, formerly classified as G. difficilis, and (D) season, for only G. fortis and G. fuliginosa. Island was not significant, even when grouping Santa Cruz with close neighbors to boost sampling power (ANOSIM p = 0.2), thus in controlling for species and island, season is still significant. Taxonomic (OTU) clustering is at 97% identity and abundance weighted by taking the fourth-root of the OTU relative abundance in each finch. Ellipses represent 90% confidence windows following a multivariate t-distribution. (PDF 909 kb) [file 40168_2018_555_MOESM6_ESM.pdf]
