# Supplementary material for: The gut of the finch: uniqueness of the gut microbiome of the Galápagos vampire finch
Source: Microbiome. 2018 Sep 19;6:167. doi: 10.1186/s40168-018-0555-8 (PMC6146768; doi:10.1186/s40168-018-0555-8)
Supplement: Supplementary file 4 — Additional information regarding the observations of finch feeding behavior, core microbiome results, and microbiome differences between vampire finches on Darwin and Wolf islands. (DOCX 20 kb) [file 40168_2018_555_MOESM4_ESM.docx]

***Processing of MiSeq data***

Raw sequence data for all 113 samples in this study (plus an additional 13 cloacal samples and 1 *C. pallida* sample) can be accessed in the Sequence Read Archive under accession number SRP130314. A sequential list of the in-house QIIME.1.8.0 commands used to process the data is given below.

1. Joining paired ends to generate contigs

join_paired_ends.py -f <sample_name_R1.fastq> -r <sample_name_R2.fastq> -m fastq-join -j 50 -p 8 -o <sample_name>_joined/

2. Quality trimming, converting from fastq to fasta format

split_libraries_fastq.py -i <sample_name>_joined/fastqjoin.join.fastq -o <sample_name>_trimmed/ -m dummy_mapping.txt --sample_id <sample_name> -q 29 -n 0 --barcode_type 'not-barcoded' --store_qual_scores

3. Concatenating all sequences from all samples

cat *checked/uchime_nonchimeras.fna > all_seqs_all_samples.fasta

4. Picking OTUs at 97% similarity (NB: 99% was also run)

pick_otus.py -i all_seqs_all_samples.fasta -s 0.97 -o uclust_picked_otus_97/

5. Picking representative sequences for each OTU

pick_rep_set.py -i uclust_picked_otus_97/all_seqs_all_samples_otus.txt -f all_seqs_all_samples.fasta -m most_abundant

6. Assigning taxonomy for each OTU

assign_taxonomy.py -i all_seqs_all_samples.fasta_rep_set.fasta -t SSURef_NR99_119_SILVA_14_07_14_opt_SACqiime.tax -r SSURef_NR99_119_SILVA_14_07_14_opt_SACqiime.fasta --uclust_similarity 0.9 --uclust_max_accepts 10 --uclust_min_consensus_fraction 0.90 -o uclust_picked_otus_97/uclust_taxa_0.9_10_0.90/

7. Making OTU table in biom format

make_otu_table.py -i uclust_picked_otus_97/all_seqs_all_samples_otus.txt -t uclust_picked_otus_97/uclust_taxa_0.9_10_0.90/all_seqs_all_samples.fasta_rep_set_tax_assignments.txt -o uclust_picked_otus_97/uclust_taxa_0.9_10_0.90/OTU_table_Silva_119_all_seqs.biom

8. Removing singleton OTUs from the dataset

filter_otus_from_otu_table.py -i uclust_picked_otus_97/uclust_taxa_0.9_10_0.90/OTU_table_Silva_119_all_seqs.biom -n 2 -o uclust_picked_otus_97/uclust_taxa_0.9_10_0.90/OTU_table_singletonfiltered.biom

9. Filtering known PCR contaminant taxa from the dataset by taxonomic name

filter_taxa_from_otu_table.py -i uclust_picked_otus_97/uclust_taxa_0.9_10_0.90/OTU_table_singletonfiltered.biom -o uclust_picked_otus_97/uclust_taxa_0.9_10_0.90/OTU_table_singleton_taxafiltered.biom -n Eukaryota

10. Generating Excel-readable OTU table with taxonomic information for use in R (*employs an in-house perl command written by Dr. Connor Skennerton)

join_otu_repset.pl uclust_picked_otus_97/uclust_taxa_0.9_10_0.90/OTU_table_singleton_taxafiltered.biom all_seqs_all_samples.fasta_rep_set.fasta > OTU_table_wTaxa_wSeqs_97.txt

11. Counting number of remaining sequences per sample to identify the level for rarefaction (found to be 9,878 sequences/sample, equal to the smallest sample, Finch-142)

biom summarize-table -i uclust_picked_otus_97/uclust_taxa_0.9_10_0.90/OTU_table_singleton_taxafiltered.biom -o OTU_table_singleton_taxafiltered.txt

***Diversity Analyses***

1. Making an OTU Multiple-Sequence Alignment

From the results of Step 5 above (pick_rep_set.py), the .fasta sequence file was uploaded to MAFFT Version 7 Online (Katoh and Standley 2013 [[*Molecular Biology and Evolution* **30**:772-780](http://mbe.oxfordjournals.org/content/30/4/772)]) to construct a multiple-sequence alignment selecting the FFT-NS-2 option (fast but rough):

mafft --reorder --memsavetree --retree 2 input

2. Generating a phylogenetic tree

In QIIME.1.8.0

make_phylogeny.py -i $PWD/aligned.fasta -o $PWD/rep_phylo.tre

3. Core Diversity Analyses

core_diversity_analyses.py -i $PWD/otu_table.biom -o $PWD/core_output -m $PWD/map.txt -c Species,Island,Season -t $PWD/rep_phylo.tre -e 9878 --suppress_beta_diversity

***DADA2 Processing***

The same 127 samples were processed using the DADA2 pipeline adapted from Callahan (2016) and the Dada2 Pipeline Tutorial v1.6 (http://benjjneb.github.io/dada2/tutorial.html).

1. Load Files

library(dada2); packageVersion("dada2")

library(ShortRead); packageVersion("ShortRead")

library(ggplot2); packageVersion("ggplot2")

path <- "~/wherefastqfilesare"

fns <- list.files(path)

fns

fastqs <- fns[grepl(".fastq$", fns)]

fastqs <- sort(fastqs) # Sort ensures forward/reverse reads are in same order

fnFs <- fastqs[grepl("_R1", fastqs)] # Just the forward read files

fnRs <- fastqs[grepl("_R2", fastqs)] # Just the reverse read files

2. Get sample names, assuming files named as so: SAMPLENAME_XXX.fastq

sample.names <- sapply(strsplit(fnFs, "_"), `[`, 1)

# Specify the full path to the fnFs and fnRs

fnFs <- file.path(path, fnFs)

fnRs <- file.path(path, fnRs)

plotQualityProfile(fnFs[[1]])

plotQualityProfile(fnFs[[2]])

plotQualityProfile(fnRs[[1]])

plotQualityProfile(fnRs[[2]])

3. Make directory and filenames for the filtered fastqs and filter

filt_path <- file.path(path, "filtered")

if(!file_test("-d", filt_path)) dir.create(filt_path)

filtFs <- file.path(filt_path, paste0(sample.names, "_F_filt.fastq.gz"))

filtRs <- file.path(filt_path, paste0(sample.names, "_R_filt.fastq.gz"))

# Filter

for(i in seq_along(fnFs)) {

fastqPairedFilter(c(fnFs[i], fnRs[i]), c(filtFs[i], filtRs[i]),

truncLen=c(220,150),

maxN=0, maxEE=c(1,1), truncQ=2, rm.phix=TRUE,

compress=TRUE, verbose=TRUE)

}

derepFs <- derepFastq(filtFs, verbose=TRUE)

derepRs <- derepFastq(filtRs, verbose=TRUE)

# Name the derep-class objects by the sample names

names(derepFs) <- sample.names

names(derepRs) <- sample.names

dadaFs.lrn <- dada(derepFs, err=NULL, selfConsist = TRUE, multithread=TRUE)

errF <- dadaFs.lrn[[1]]$err_out

dadaRs.lrn <- dada(derepRs, err=NULL, selfConsist = TRUE, multithread=TRUE)

errR <- dadaRs.lrn[[1]]$err_out

plotErrors(dadaFs.lrn[[1]], nominalQ=TRUE)

4. Sample Inference

dadaFs <- dada(derepFs, err=errF, multithread=TRUE)

dadaRs <- dada(derepRs, err=errR, multithread=TRUE)

dadaFs[[1]]

5. Merged Paired Reads

mergers <- mergePairs(dadaFs, derepFs, dadaRs, derepRs, verbose=TRUE)

# Inspect the merger data.frame from the first sample

head(mergers[[1]])

6. Make Sequence Table

seqtab <- makeSequenceTable(mergers)

dim(seqtab)

table(nchar(getSequences(seqtab))) #shows distribution of lengths

# cut to only lengths between 250-256 following inspection

seqtab2 <- seqtab[,nchar(colnames(seqtab)) %in% seq(250,256)]

7. Remove chimeras

seqtab2.nochim <- removeBimeraDenovo(seqtab2, verbose=TRUE)

dim(seqtab2.nochim)

sum(seqtab2.nochim)/sum(seqtab2)

8. Assign taxonomy using Silva Training Set (https://www.arb-silva.de/)

ref_fasta <- "silva_nr_v123_train_set.fa.gz"

taxtab <- assignTaxonomy(seqtab2.nochim, refFasta = ref_fasta)

colnames(taxtab) <- c("Kingdom", "Phylum", "Class", "Order", "Family", "Genus")

write.csv(taxtab, file=“taxtab.csv”)

write.csv(seqtab2.nochim, file=“datable.csv”)

***NMDS, ANOSIM, and SIMPER tests in R***

Generic examples of the three commands are given below, including the options employed for this study. All commands require installation of the ‘vegan’ package in R, as well as two dependencies: ‘lattice’ and ‘permute’. For this study, the following package versions were employed:

‘phyloseq’: v1.25-2

‘vegan’: v2.4-5

‘lattice’: v0.20-35

‘permute’: v0.9-4

1. NMDS

NMDS_analysis=metaMDS(transformed_data,distance="bray",k=2,trymax=100,engine=c("monoMDS"),autotransform=FALSE)

2. ANOSIM

ANOSIM_analysis=with(sample_metadata,anosim(transformed_data,Season,permutations=999,distance="bray"))

3. Adonis

Adonis_analysis=adonis(transformed_data~Season,data=sample_metadata,permutations=999,method="bray")

4. SIMPER

SIMPER_analysis=with(sample_metadata,simper(transformed_data,Season))

***Data Visualization***

The following additional R packages were used:

‘readxl’: v1.0.0

‘ggplot2’: v2.2.1
