# Supplementary material for: The gut of the finch: uniqueness of the gut microbiome of the Galápagos vampire finch
Source: Microbiome. 2018 Sep 19;6:167. doi: 10.1186/s40168-018-0555-8 (PMC6146768; doi:10.1186/s40168-018-0555-8)
Supplement: Supplementary file 5 — Table S3. Analysis of similarities (ANOSIM) and PERMANOVA (Adonis) significance of each grouping within each dataset subdivision (as described in the Methods section) for weighted (to the 4th-root) and unweighted (presence-absence) relative abundance. OTUs were calculated in QIIME at 97%. Significant statistics (p < 0.05) are highlighted in yellow. Adonis models used the sample size-balanced variable first when multiple variables were tested. Bars in cells indicate that variance-partitioning was impossible, often due to the factor overlapping with the first factor (ex. In 12c, latitude overlaps heavily with island, with island explaining all and more of the variation attributable to latitude). Dataset subdivisions labeled with a ** indicate small sample size; results are listed as they may show a contrasting trend requiring further investigation. Grayed-out regions in the table are either untestable because the dataset subdivision only has one category in that factor (dark gray) or were not tested because they are redundant with other data subdivisions that have better balance or sample size (light gray). Data subdivisions are color-coded by the factor tested (green = season, blue = diet, red = vampire, yellow = island, purple = species). (PDF 129 kb) [file 40168_2018_555_MOESM5_ESM.pdf]

Table S3.

A.

		Season				Diet				Vampire			
		ANOSIM		Adonis		ANOSIM		Adonis		ANOSIM		Adonis	
		R	*p*	R^2	*p*	*R*	*p*	R^2	*p*	*R*	*p*	R^2	*p*
1. Full Dataset *N = 113*	Weighted Bray-Curtis	0.27	0.001	0.041	0.001	0.15	0.001	0.010	0.056	0.23	0.001	0.051	0.001
	Unweighted Bray-Curtis	0.20	0.001	0.038	0.001	0.19	0.001	0.009	0.12	0.31	0.001	0.057	0.001
	Weighted UniFrac	0.30	0.001	0.073	0.001	0.12	0.001	0.008	0.18	0.16	0.001	0.060	0.001
	Unweighted UniFrac	0.21	0.001	0.029	0.001	0.26	0.001	0.012	0.013	0.38	0.001	0.050	0.001
2. November samples *N = 60*	Weighted Bray-Curtis					0.33	0.001	0.017	0.19	0.35	0.001	0.11	0.001
	Unweighted Bray-Curtis					0.32	0.001	0.017	0.21	0.35	0.001	0.12	0.001
	Weighted UniFrac					0.30	0.001	0.016	0.26	0.29	0.001	0.14	0.001
	Unweighted UniFrac					0.41	0.001	0.020	0.064	0.43	0.001	0.10	0.001
3a. No *G. septentrionalis,* balanced by season *N = 82*	Weighted Bray-Curtis	0.17	0.001	0.056	0.001	-0.07	0.95	0.018	0.076				
	Unweighted Bray-Curtis	0.14	0.001	0.049	0.001	-0.05	0.88	0.017	0.15				
	Weighted UniFrac	0.17	0.001	0.078	0.001	-0.05	0.87	0.015	0.28				
	Unweighted UniFrac	0.13	0.001	0.036	0.001	-0.03	0.75	0.02	0.01				
3b. No *G. septentrionalis,* balanced season & diet *N = 40*	Weighted Bray-Curtis	0.17	0.001	0.068	0.002	0.019	0.32	0.032	0.24				
	Unweighted Bray-Curtis	0.15	0.002	0.063	0.002	0.022	0.27	0.031	0.27				
	Weighted UniFrac	0.15	0.009	0.087	0.004	0.008	0.34	0.028	0.31				
	Unweighted UniFrac	0.14	0.002	0.052	0.001	0.062	0.10	0.034	0.053				
4. Herbivorous finches, balanced by season *N = 50*	Weighted Bray-Curtis	0.21	0.001	0.074	0.001								
	Unweighted Bray-Curtis	0.17	0.001	0.068	0.001								
	Weighted UniFrac	0.24	0.001	0.11	0.001								
	Unweighted UniFrac	0.16	0.001	0.050	0.001								
5. Insectivorous finches, balanced by season *N = 20*	Weighted Bray-Curtis	0.076	0.17	0.077	0.12								
	Unweighted Bray-Curtis	0.072	0.18	0.071	0.16								
	Weighted UniFrac	0.042	0.23	0.074	0.18								
	Unweighted UniFrac	0.11	0.09	0.075	0.082								
6a. *G. fuliginosa*, balanced by season *N = 22*	Weighted Bray-Curtis	0.38	0.001	0.12	0.002								
	Unweighted Bray-Curtis	0.38	0.001	0.12	0.002								
	Weighted UniFrac	0.45	0.001	0.17	0.005								
	Unweighted UniFrac	0.31	0.002	0.086	0.006								
7a. *G. fuliginosa* & *G. fortis*, balanced by season N = 30	Weighted Bray-Curtis	0.28	0.001	0.11	0.001								
	Unweighted Bray-Curtis	0.26	0.001	0.11	0.001								
	Weighted UniFrac	0.35	0.001	0.18	0.001								
	Unweighted UniFrac	0.23	0.001	0.078	0.001								
8. *G. fuliginosa* on San Cristobal Island, balanced by season *N = 14*	Weighted Bray-Curtis	0.46	0.003	0.20	0.004								
	Unweighted Bray-Curtis	0.43	0.003	0.19	0.003								
	Weighted UniFrac	0.47	0.007	0.27	0.006								
	Unweighted UniFrac	0.40	0.005	0.15	0.005								
9. No *G. septentrionalis*, dry season, balanced by diet N = 32	Weighted Bray-Curtis					0.018	0.30	0.036	0.32				
	Unweighted Bray-Curtis					0.011	0.38	0.036	0.36				
	Weighted UniFrac					0.015	0.33	0.044	0.21				
	Unweighted UniFrac					0.028	0.24	0.039	0.22				
10. No *G. septentrionalis*, wet season, balanced diet N = 20	Weighted Bray-Curtis					0.012	0.43	0.062	0.33				
	Unweighted Bray-Curtis					0.044	0.28	0.063	0.28				
	Weighted UniFrac					0.013	0.40	0.054	0.42				
	Unweighted UniFrac					0.10	0.12	0.075	0.075				
11. Herbivores and insectivores, Santa Cruz, wet season, unbalanced** N = 10	Weighted Bray-Curtis					0.75	0.005	-	-				
	Unweighted Bray-Curtis					0.42	0.017	-	-				
	Weighted UniFrac					0.24	0.079	-	-				
	Unweighted UniFrac					0.35	0.019	-	-				
12a. Ground finches during the dry season, balanced by diet** N = 18	Weighted Bray-Curtis					0.11	0.16	0.087	0.067				
	Unweighted Bray-Curtis					0.14	0.057	0.095	0.031				
	Weighted UniFrac					0.15	0.093	0.14	0.020				
	Unweighted UniFrac					0.18	0.024	0.093	0.007				
13a. Dry season finches, balanced by vampire N = 62	Weighted Bray-Curtis					0.32	0.001	0.016	0.35	0.34	0.001	0.10	0.001
	Unweighted Bray-Curtis					0.32	0.001	0.016	0.42	0.33	0.001	0.11	0.001
	Weighted UniFrac					0.25	0.001	0.018	0.34	0.23	0.001	0.090	0.001
	Unweighted UniFrac					0.38	0.001	0.017	0.27	0.41	0.001	0.095	0.001
13b. Dry season finches, balanced by vampire N = 20	Weighted Bray-Curtis					0.33	0.001	0.048	0.49	0.38	0.003	0.15	0.003
	Unweighted Bray-Curtis					0.36	0.001	0.051	0.46	0.39	0.002	0.15	0.003
	Weighted UniFrac					0.28	0.001	0.056	0.36	0.28	0.019	0.14	0.016
	Unweighted UniFrac					0.42	0.001	0.055	0.35	0.45	0.003	0.14	0.016
14. Feather isotope finches, dry season, balanced by vampire N = 30	Weighted Bray-Curtis									0.30	0.001	0.14	0.001
	Unweighted Bray-Curtis									0.29	0.001	0.14	0.001
	Weighted UniFrac									0.18	0.002	0.12	0.002
	Unweighted UniFrac									0.34	0.001	0.12	0.002
15. Three ex-"*G. difficilis*", balanced by vampire N = 18	Weighted Bray-Curtis									0.57	0.001	0.20	0.001
	Unweighted Bray-Curtis									0.58	0.001	0.22	0.001
	Weighted UniFrac									0.46	0.001	0.23	0.001
	Unweighted UniFrac									0.69	0.001	0.19	0.001

B.

		Species				Island				Latitude			
		ANOSIM		Adonis		ANOSIM		Adonis		ANOSIM		Adonis	
		R	***p***	***R^2***	***p***	***R***	***p***	***R^2***	***p***	***R***	***p***	***R^2***	***p***
1. Full Dataset	Weighted Bray-Curtis	0.27	0.001	0.077	0.003	0.21	0.001	0.078	0.001	0.14	0.001	0.048	0.001
N = 113	Unweighted Bray-Curtis	0.27	0.001	0.076	0.002	0.24	0.001	0.077	0.001	0.16	0.001	0.050	0.001
	Weighted UniFrac	0.24	0.001	0.073	0.019	0.17	0.001	0.082	0.001	0.097	0.001	0.051	0.001
	Unweighted UniFrac	0.34	0.001	0.080	0.001	0.27	0.001	0.062	0.001	0.19	0.001	0.045	0.001
2. November samples	Weighted Bray-Curtis	0.40	0.001	0.11	0.004	0.36	0.001	0.072	0.001	-0.01	0.50	0.024	0.028
N = 60	Unweighted Bray-Curtis	0.38	0.001	0.11	0.001	0.38	0.001	0.079	0.001	-0.01	0.56	0.024	0.027
	Weighted UniFrac	0.36	0.001	0.090	0.051	0.35	0.001	0.086	0.001	-0.04	0.62	0.025	0.036
	Unweighted UniFrac	0.45	0.001	0.098	0.003	0.43	0.001	0.086	0.001	0.058	0.24	0.026	0.009
3a. No *G.*	Weighted Bray-Curtis	-0.01	0.52	0.15	0.001	0.052	0.090	0.070	0.001				
septentrionalis,	Unweighted Bray-Curtis	0.030	0.30	0.15	0.001	0.051	0.092	0.064	0.002				
balanced by season	Weighted UniFrac	-0.01	0.54	0.16	0.001	0.037	0.15	0.072	0.003				
N = 82	Unweighted UniFrac	0.070	0.11	0.14	0.001	0.034	0.17	0.060	0.002				
6a. *G. fuliginosa*,	Weighted Bray-Curtis					0.10	0.21	0.12	0.05				
balanced by season	Unweighted Bray-Curtis					0.10	0.20	0.11	0.09				
N = 22	Weighted UniFrac					0.06	0.30	0.10	0.21				
	Unweighted UniFrac					0.11	0.15	0.11	0.08				
6b. *G. fuliginosa*,	Weighted Bray-Curtis					0.35	0.07	0.23	0.06				
balanced by island*	Unweighted Bray-Curtis					0.37	0.09	0.23	0.07				
N = 8	Weighted UniFrac					0.35	0.10	0.25	0.11				
	Unweighted UniFrac					0.30	0.13	0.19	0.11				
7b. *G. fuliginosa* & *G.*	Weighted Bray-Curtis	0	0.48	0.048	0.29								
fortis,	Unweighted Bray-Curtis	0.020	0.36	0.052	0.21								
balanced by species	Weighted UniFrac	0.049	0.22	0.057	0.17								
N = 22	Unweighted UniFrac	0.040	0.27	0.070	0.20								
7c. *G. fuliginosa* & *G.*	Weighted Bray-Curtis					0.060	0.15	0.060	0.066				
fortis,	Unweighted Bray-Curtis					0.031	0.28	0.054	0.12				
balanced by island	Weighted UniFrac					0.038	0.22	0.045	0.18				
N = 24	Unweighted UniFrac					0.058	0.20	0.054	0.11				
12b. Ground finches	Weighted Bray-Curtis	0.33	0.016	0.37	0.008								
during the dry season,	Unweighted Bray-Curtis	0.35	0.008	0.39	0.002								
balanced by species**	Weighted UniFrac	0.32	0.027	0.41	0.010								
N = 15 (n=3 x 5 sp.)	Unweighted UniFrac	0.28	0.030	0.35	0.004								
12c. Ground finches	Weighted Bray-Curtis					0.37	0.001	0.25	0.001	0.20	0.005	-	-
during the dry season,	Unweighted Bray-Curtis					0.34	0.001	0.26	0.001	0.24	0.001	-	-
balanced by island**	Weighted UniFrac					0.42	0.001	0.35	0.001	0.22	0.005	-	-
N = 24 (n=6 x 4 islands)	Unweighted UniFrac					0.35	0.001	0.22	0.001	0.32	0.001	-	-
15. Three ex-"*G.*	Weighted Bray-Curtis	0.53	0.002	-	-	0.60	0.001	0.17	0.015				
difficilis",	Unweighted Bray-Curtis	0.50	0.002	-	-	0.60	0.001	0.17	0.018				
balanced by vampire	Weighted UniFrac	0.47	0.003	-	-	0.49	0.001	0.19	0.017				
N = 18	Unweighted UniFrac	0.50	0.002	-	-	0.56	0.001	0.13	0.087				
16. Herbivorous finches,	Weighted Bray-Curtis	0.28	0.025	0.30	0.03								
Santa Cruz, dry season,	Unweighted Bray-Curtis	0.41	0.003	0.32	0.008								
~balanced species**	Weighted UniFrac	0.12	0.20	0.24	0.28								
N = 10	Unweighted UniFrac	0.29	0.017	0.25	0.14								
17. *G. septentrionalis,*	Weighted Bray-Curtis					0.16	0.001	0.093	0.001				
balanced by island	Unweighted Bray-Curtis					0.21	0.001	0.11	0.001				
N = 30	Weighted UniFrac					0.18	0.002	0.11	0.001				
	Unweighted UniFrac					0.23	0.001	0.092	0.001				
18. *G. fuliginosa* & *G.*	Weighted Bray-Curtis	0.16	0.18	0.09	0.75	-0.11	0.73	0.13	0.38				
fortis, wet season,	Unweighted Bray-Curtis	0.15	0.16	0.10	0.59	0.15	0.16	0.13	0.37				
balanced by island**	Weighted UniFrac	0.13	0.22	0.14	0.26	0.13	0.22	0.13	0.35				
N = 10	Unweighted UniFrac	0.16	0.18	0.12	0.43	0.16	0.18	0.13	0.28				
19. *G. fuliginosa* & *G.*	Weighted Bray-Curtis	0.25	0.10	0.10	0.05	0.32	0.004	0.15	0.002				
fortis, dry season,	Unweighted Bray-Curtis	0.25	0.11	0.11	0.03	0.29	0.005	0.16	0.002				
balanced by island**	Weighted UniFrac	0.16	0.19	0.067	0.28	0.43	0.010	0.22	0.008				
N = 14	Unweighted UniFrac	0.23	0.11	0.089	0.11	0.26	0.014	0.13	0.005				
