# Supplementary material for: The gut of the finch: uniqueness of the gut microbiome of the Galápagos vampire finch
Source: Microbiome. 2018 Sep 19;6:167. doi: 10.1186/s40168-018-0555-8 (PMC6146768; doi:10.1186/s40168-018-0555-8)
Supplement: Supplementary file 7 — Table S4. Description of dataset subdivisions for analysis of individual factors without confounding factors, taken by randomly sub-setting a sub-category of the dataset subdivision where appropriate, as indicated. The influence of season was analyzed using data subdivisions 1 and 3-8, thereby controlling for different species/island/diet groups and the singularity of the vampire finches. Diet was assessed with dataset subdivisions 9-12 by comparing all finch species sampled with the exception of the vampire finches binned into diet categories of herbivorous (including seeds, cactus, and plant matter) and omnivorous (including insects), with control for season and island embedded in the various subsets. Seed-eaters are not generally considered herbivores, thus these groupings were designed broadly to encompass plant matter versus other dietary foodstuffs. The uniqueness of sanguivory was tested in dataset subdivisions 13-15, where season, sample size, and phylogeny (through nearest relatives) were considered as well as a comparison with other finches whose feather isotopes were measured. The role of finch species grouping was analyzed in dataset subdivisions 3, 7, 12, 16, 18, and 19 to remove the confounding influence of the extreme diet of G. septentrionalis and to extract the largest groupings while avoiding potential biogeographical (island) and season bias and to assess the ground finches alone. The influence of island was assessed in dataset subdivisions 1-3, 6, 7, 12, and 17-19, with Santa Cruz, Santa Fé, and North Seymour sometimes grouped together as “Santa Cruz +” since these islands are in close geographic proximity (Fig. 1). In some cases (dataset subdivisions 1, 2, and 12), latitude was assessed, as the northern, more isolated islands of Pinta, Genovesa, Wolf, and Darwin versus the remaining islands of the Galápagos. (DOCX 135 kb) [file 40168_2018_555_MOESM7_ESM.docx]

| **Dataset** | **Description** | **Sample Size** |
| --- | --- | --- |
| 1. Full dataset | To provide an overview of the data: all samples | 113 |
| 2. November | To account for the possibility of month-to-month sampling variation | 60 |
| 3. Excluding *G. septentrionalis* | To disentangle the vampire finch factor for assessing the significance of other factors |  |
| a. Balanced by season | 41 finches from each of the wet and dry seasons (not subset) | 82 |
| b. Balanced season & diet | 10 herbivorous/dry season (random subset from 31), 10 herbivorous/wet (random subset from 25), 10 insectivorous/dry (random subset from 16), and 10 insectivorous/wet | 40 |
| 4. Herbivores | To test season accounting for diet, 25 wet-season and 25 (random subset from 31) dry-season | 50 |
| 5. Insectivores | To test season accounting for diet, 10 wet-season and 10 (random subset from 16) dry-season | 20 |
| 6. *G. fuliginosa* | The small-beaked ground finch, an herbivore (seeds) sampled across the wet/dry seasons and San Cristóbal/Santa Cruz Islands |  |
| a. Balanced by season | To test season accounting for species, 11 dry-season and 11 (random subset from 16) wet-season | 22 |
| b. Balanced by island | To test island accounting for species and season, during the dry season only, 4 Santa Cruz and 4 (subset from 7) San Cristóbal | 8^a^ |
| 7. *G. fuliginosa* & *G. fortis* | The small- and medium-beak ground finch (both seed-eaters), sampled across the wet/dry seasons and San Cristóbal/Santa Cruz Islands |  |
| a. Balanced by season | To test season roughly accounting for species, 15 dry-season and 15 (random subset from 18) wet-season | 30 |
| b. Balanced by species | To confirm the species are not distinct to test (a) and (c), 11 *G. fortis* and 11 (subset from 22) *G. fuliginosa* | 22 |
| c. Balanced by island | To test island roughly accounting for species, 12 Santa Cruz, Santa Fé, and North Seymour island group and 12 (random subset from 21) San Cristóbal | 24 |
| 8. *G. fuliginosa* on San Cristóbal Island | To test season accounting for species and island, 7 dry-season and 7 (random subset from 8) wet-season | 14 |
| 9. No *G. septentrionalis*, dry season | To test diet accounting for sanguivory and season, 16 insectivorous and 16 (random subset from 25) herbivorous finches | 32 |
| 10. No *G. septentrionalis*, wet season | To test diet accounting for sanguivory and season, 10 insectivorous and 10 (random subset from 31) herbivorous finches | 20 |
| 11. Herbivores and insectivores, Santa Cruz, wet season | To test diet accounting for sanguivory, island, and season, 6 herbivorous (3 *Patyspiza crassirostris*, 1 *G. fuliginosa*, 1 *G. fortis*, and 1 *G. scandens*) and 4 insectivorous (C. parvulus) finches | 10 |
| 12. Ground finches during the dry season | *G. difficilis, G. acutirostris, G. magnirostris, G. fortis,* and *G. fuliginosa* |  |
| a. balanced by diet | To test diet accounting for genus and season, 9 insectivorous (*G. difficilis, G. acutirostris*) and 9 (random subset from 20) herbivorous finches (*G. magnirostris, G. fortis, G. fuliginosa*) | 18^b^ |
| b. balanced by species | To test species within a famous genus accounting for season, 3 *Geospiza fuliginosa* (random subset from 11), 3 *G. fortis* (random subset from 4), 3 *G. magnirostris* (random subset from 5), 3 *G. difficilis,* and 3 *G. acutirostris* (random subset from 6) | 12^a^ |
| c. balanced by island | To test island accounting for season and genus, from Pinta (n = 6), Genovesa (n = 6), Santa Cruz (n = 6), and San Cristóbal (n = 6) | 24 |
| 13. Dry season finches |  |  |
| a. balanced by vampire | To test sanguivory accounting for season, 31 *G. septentrionalis* and 31 other finches | 62 |
| b. balanced by vampire (small sample) | To compare to other small-sample data sets, 10 (random subset from 31) *G. septentrionalis* and 10 (random subset from 31) other finches | 20 |
| 14. Feather isotope finches | To test diet in comparison with isotope data (Figure 3), 15 *G. fuliginosa* and *G. fortis* and 15 (random subset from 31) *G. septentrionalis* during the dry season | 30 |
| 15. Three ex-“*G. difficilis*” | Three finch species formerly known as *Geospiza difficilis,* including 3 *G. difficilis* on Pinta, 6 *G. acutirostris* on Genovesa, and 9 (random subset from 31) *G. septentrionalis* | 18 |
| 16. Santa Cruz Island dry season herbivorous species | To test species accounting for island, season, and diet, 4 *G. fuliginosa,* 3 *G. fortis,* and 3 *G. scandens* | 10^a^ |
| 17. *G. septentrionalis* | To test island in the vampire finches, during the dry season, 15 from Wolf and 15 (random subset from 16) from Darwin Island | 30 |
| 18. *G. fortis* and *G. fuliginosa* during the wet season | To test island during the wet season roughly accounting for species, 5 from Santa Cruz, Santa Fé, and North Seymour island group and 5 (subset from 13) from San Cristóbal Island | 10^a^ |
| 19. *G. fortis* and *G. fuliginosa* during the dry season | To test island during the dry season roughly accounting for species, 7 from Santa Cruz, Santa Fé, and North Seymour island group and 7 (subset from 8) from San Cristóbal Island | 14^a^ |
|  |  |  |

*^a^small sample size: results are included as tentative and require further investigation through deeper sampling*

*^b^other factors convolute the interpretation of the diet factor in this case: results are included as tentative and require further investigation*
